# Supplementary material for: Systematic Discovery of Pathogen Effector Functions across Human Pathogens and Pathways
Source: bioRxiv. 2025 Nov 17:2025.11.17.687821. Preprint. [Version 1] doi: 10.1101/2025.11.17.687821 (PMC12667889; doi:10.1101/2025.11.17.687821)
Supplement: Supplement 7 [file NIHPP2025.11.17.687821v1-supplement-7.pdf]

bioRxiv preprint doi: https://doi.org/10.1101/2025.11.17.687821; this version posted November 17, 2025. The copyright holder for this preprint (which was not certified by peer review) is the author/funder, who has granted bioRxiv a license to display the preprint in perpetuity. It is made available under aCC-BY 4.0 International license.

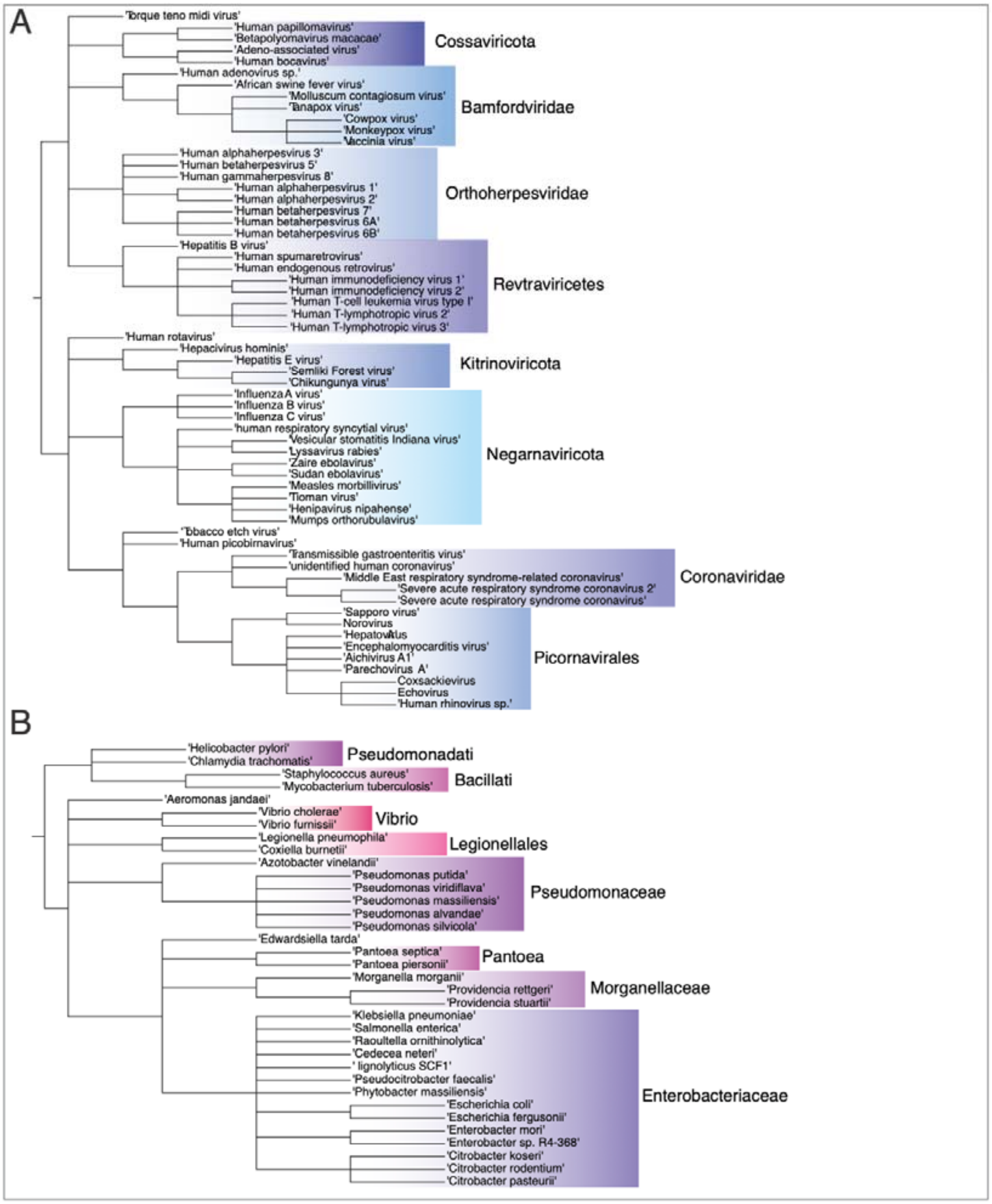

Supplementary Figure 1. Evolutionary relationships of eORFs in the eORFeome library. Phylogenetic tree of the viral (A) or bacterial (B) eORFs present in the eORFeome library.

bioRxiv preprint doi: https://doi.org/10.1101/2025.11.17.687821; this version posted November 17, 2025. The copyright holder for this preprint (which was not certified by peer review) is the author/funder, who has granted bioRxiv a license to display the preprint in perpetuity. It is made available under aCC-BY 4.0 International license.

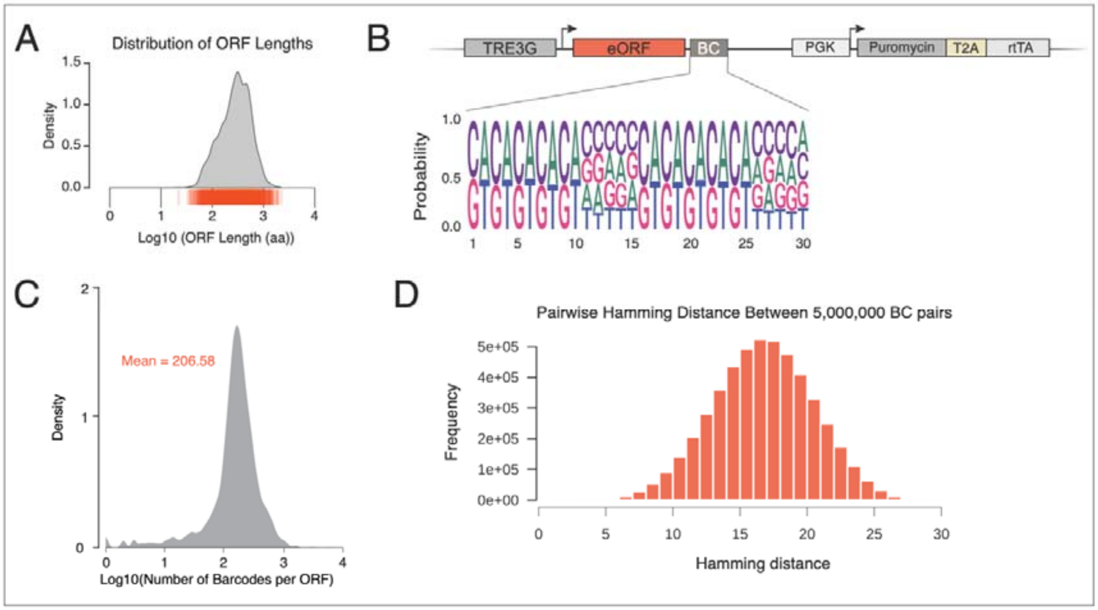

Supplementary Figure 2. Characterization of the eORFeome library. (A) Distribution of eORF lengths. The density plot is based on the Log10-transformed length (in amino acids) of each ORF in the library. The underlying orange lines marks the position of each individual eORF. (B) Top: Scheme of the lentiviral construct, detailing the arrangement of its key components: a dox-inducible TRE3G promoter drives the expression of an eORF, which is followed by a unique barcode (BC). Downstream of this, a constitutive promoter ensures the expression of both puromycin for cell selection and rtTA, which is essential for the dox-inducible activation of the TRE3G promoter. Bottom: position weight matrix that describes the semi-random nature of the 30-nucleotide barcode (BC), which consists of the pattern [(SW)×5 + N×5] repeated twice, where S represents G or C, W represents A or T, and N represents any nucleotide. (C) Density plot of the Log10 distribution of the number of barcodes per ORF, with a mean of 206.58 BC per ORF. (D) Histogram of the Hamming distance distribution, where the frequency (y-axis) of a given Hamming distance (x-axis) was calculated from 5 million randomly sampled barcode pairs from the library.

bioRxiv preprint doi: https://doi.org/10.1101/2025.11.17.687821; this version posted November 17, 2025. The copyright holder for this preprint (which was not certified by peer review) is the author/funder, who has granted bioRxiv a license to display the preprint in perpetuity. It is made available under aCC-BY 4.0 International license.

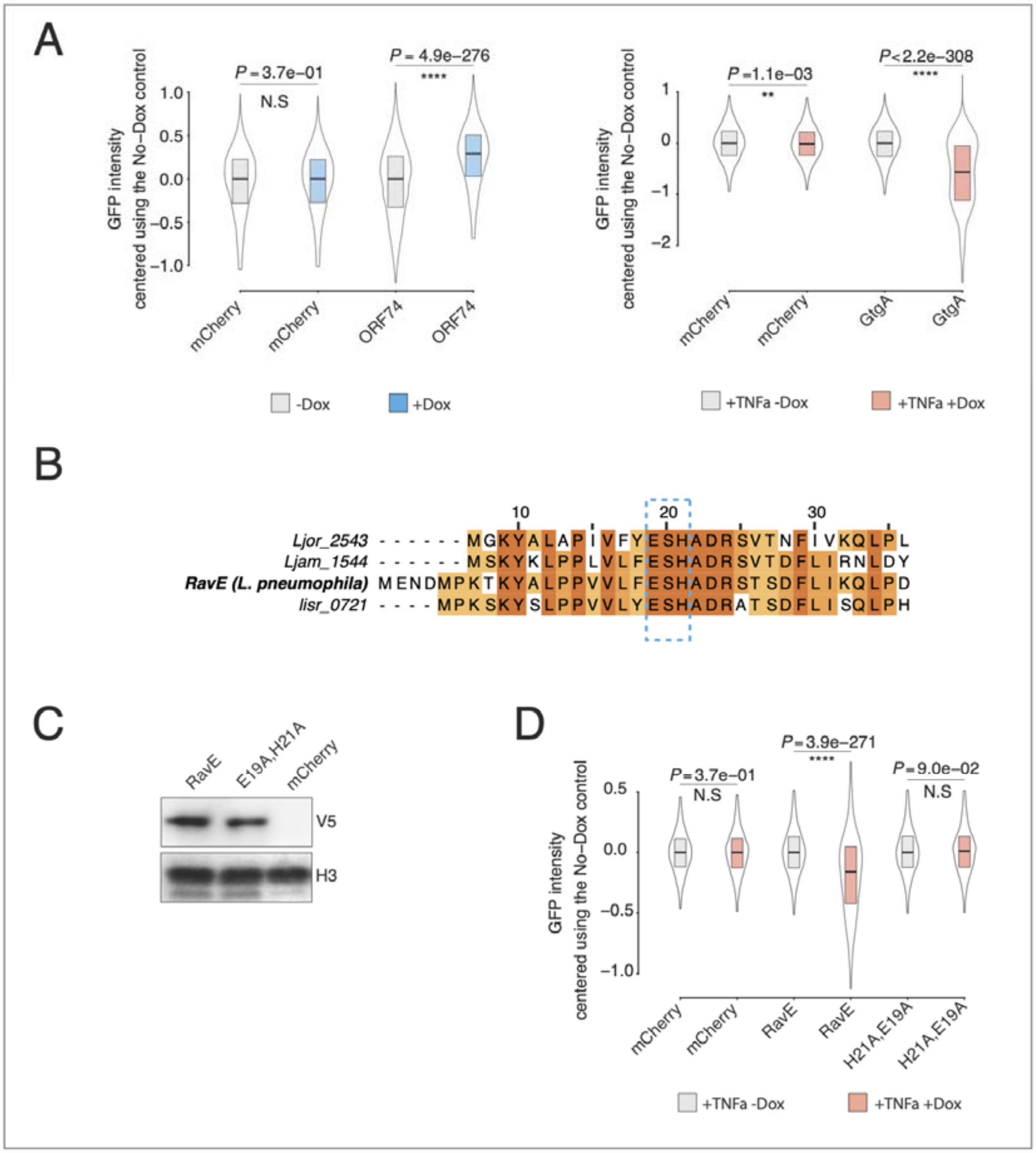

Supplementary Figure 3. Validation of eORF hits from NF-κB Screens. (A) Boxplots showing representative examples of the single-cell GFP fluorescence distribution, complementing the median-based analysis presented in Figure 2B. ORF expression was induced with dox (blue or orange) or left untreated (grey) and where specified, cells were treated with 20 ng/mL TNFα for 16 hours to stimulate NF-κB activity. The y-axis represents the Logicle-transformed GFP fluorescence, centred to the median of the corresponding no-dox control population. Boxplots display the median and interquartile range. mCherry serves as the negative control. Statistical significance was determined by comparing dox-treated versus untreated samples for each ORF. (B) Multiple sequence alignment of four RavE homologs. Conserved residues are coloured in orange. (C) Western blot analysis of cells expressing V5-tagged RavE, V5-tagged RavE E19A,H21A mutant, or an mCherry control. Lysates were immunoblotted with an anti-V5 antibody to confirm that the mutant protein is stably expressed. H3 served as a loading control. (D) Boxplots showing single-cell GFP fluorescence distributions, generated using the same analysis method described in (A). These data complement the median-based analysis presented in Figure 2J.

bioRxiv preprint doi: https://doi.org/10.1101/2025.11.17.687821; this version posted November 17, 2025. The copyright holder for this preprint (which was not certified by peer review) is the author/funder, who has granted bioRxiv a license to display the preprint in perpetuity. It is made available under aCC-BY 4.0 International license.

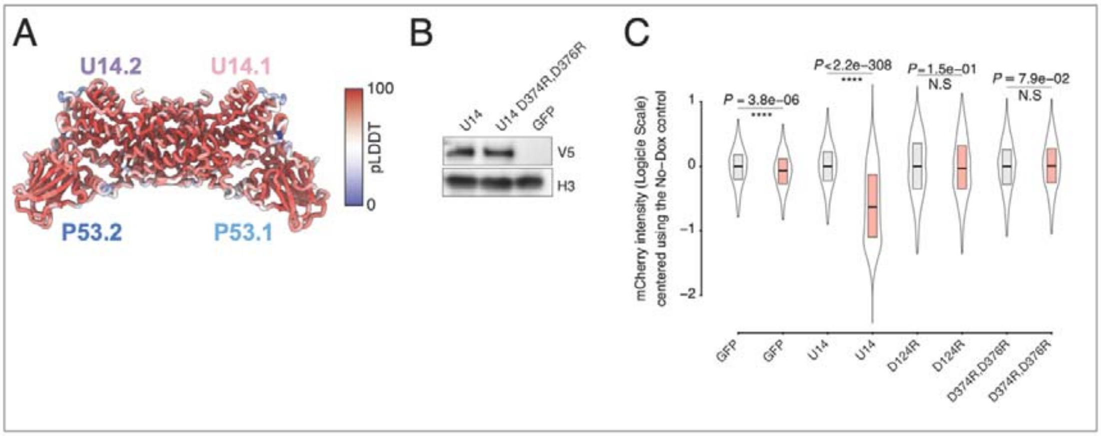

Supplementary Figure 4. Validation of U14 from HHV6A as a p53 antagonist. (A) Multiple sequence alignment of four RavE homologs. Conserved residues are coloured in orange. (B) Western blot analysis of cells expressing V5-tagged U14, V5-tagged U14 D124R mutant, or an GFP control. Lysates were immunoblotted with an anti-V5 antibody to confirm that the mutant protein is stably expressed. H3 served as a loading control. (C) Predicted local distance difference test (pLDDT) scores for the U14–p53 complex structural model generated by AlphaFold 3. (B) Western blot analysis of cells expressing V5-tagged U14, V5-tagged U14 D374R,D376R mutant, or an mCherry control. Lysates were immunoblotted with an anti-V5 antibody to confirm that the mutant protein is stably expressed. H3 served as a loading control. (C) Boxplots showing representative examples of the single-cell mCherry fluorescence distribution, complementing the median-based analysis presented in Figure 4. ORF expression was induced with dox (orange) or left untreated (grey) and where specified, cells were treated with 2.5uM nutlin for 16 hours to stimulate p53 activity. The panels show results for wild-type U14 with its inactive mutants (D124R or D374R,D376R). The y-axis represents the Logicle-transformed mCherry fluorescence, centred to the median of the corresponding no-dox control population. Boxplots display the median and interquartile range. GFP serves as the negative control. Statistical significance was determined by comparing dox-treated versus untreated samples for each ORF. *padj < 0.05, **padj < 0.01, ***padj < 0.001, **** padj < 0.0001.

bioRxiv preprint doi: https://doi.org/10.1101/2025.11.17.687821; this version posted November 17, 2025. The copyright holder for this preprint (which was not certified by peer review) is the author/funder, who has granted bioRxiv a license to display the preprint in perpetuity. It is made available under aCC-BY 4.0 International license.

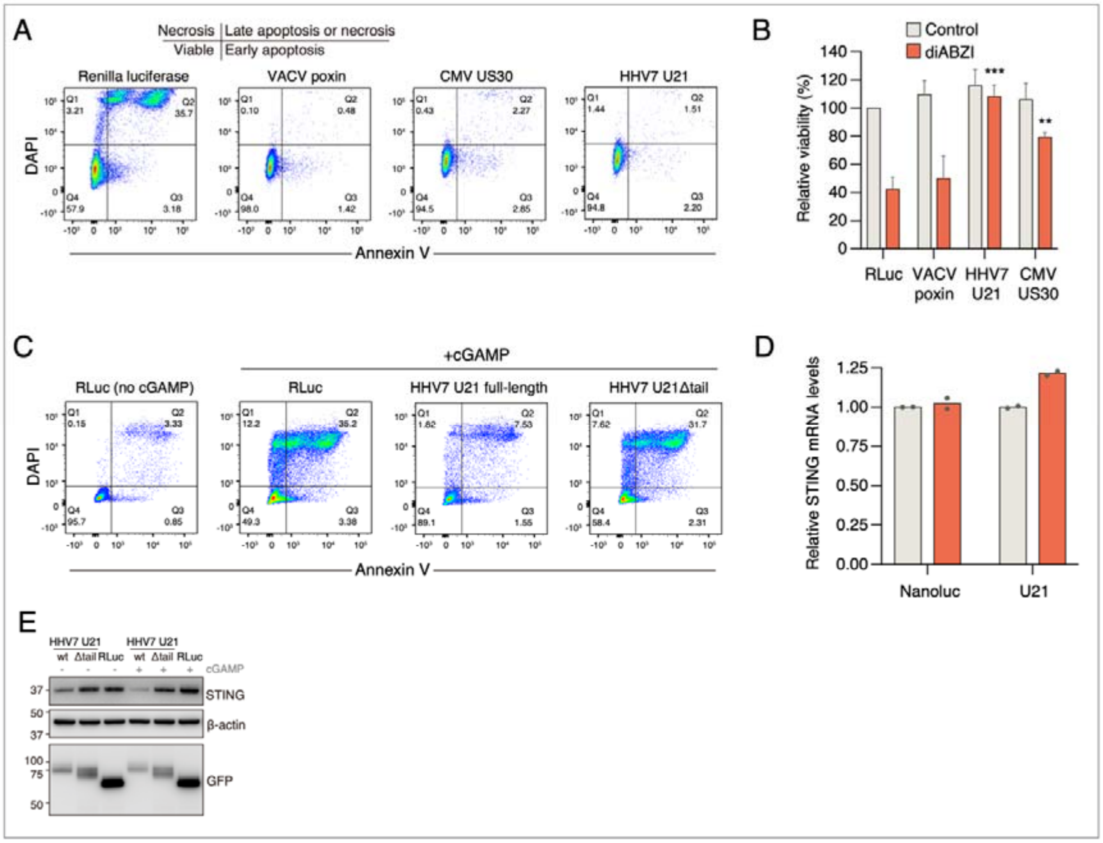

Supplementary Figure 5. Validation of U21 as a potent inhibitor of STING signaling. (A) U937 cells stably expressing dox-inducible GFP-tagged Renilla luciferase, VACV poxin, CMV US30, or HHV7 U21 were induced with dox followed by 2’,3’-cGAMP treatment and analyzed for apoptosis with Annexin V and DAPI staining. (B) The same constructs were analyzed for viability CellTiter-Glo after treating the cells with the non-nucleotide STING agonist diABZI. (C) Cells expressing full-length HHV7 U21 or U21Δtail were analyzed for apoptosis after cGAMP treatment as in (A). (D) STING mRNA levels were assessed by qRT-PCR in cells expressing Nanoluc-GFP or U21-GFP. (E) STING protein levels were analyzed by western blotting in cells expressing the indicated GFP-tagged constructs after treating the cells with cGAMP or vehicle.

bioRxiv preprint doi: https://doi.org/10.1101/2025.11.17.687821; this version posted November 17, 2025. The copyright holder for this preprint (which was not certified by peer review) is the author/funder, who has granted bioRxiv a license to display the preprint in perpetuity. It is made available under aCC-BY 4.0 International license.

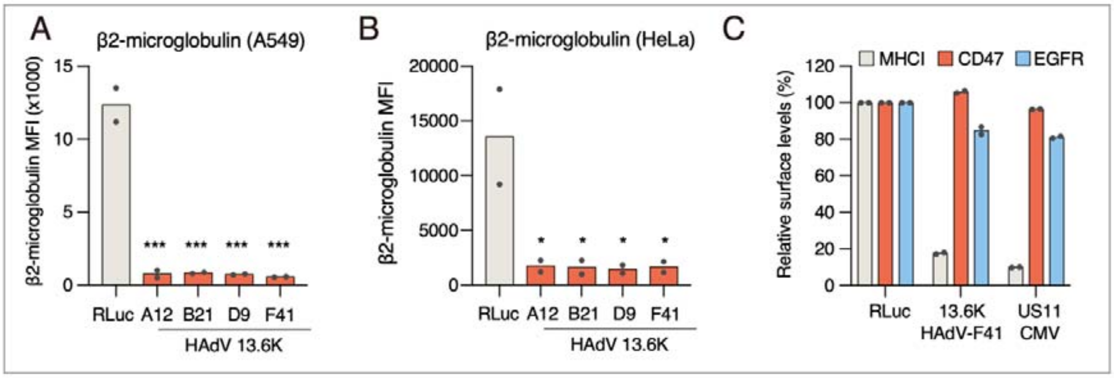

Supplementary Figure 6. Validation of 13.6K proteins as novel TAP inhibitors. (A-B) A549 cells (A) and HeLa cells (B) expressing indicated GFP-tagged constructs were assessed for cell surface β2-microglobulin levels by flow cytometry. (C) HeLa cells expressing GFP-tagged HAdV-F41 13.6K or CMV US11, a known inhibitor of MHC-I surface display, were analyzed for cell surface MHC-I, EGFR, or CD47 levels by flow cytometry.
